# Association between tacrolimus blood levels and biopsy-proven acute cellular rejection in adult heart transplant recipients

**DOI:** 10.1016/j.jhlto.2025.100373

**Published:** 2025-08-19

**Authors:** Chengliang Yang, Casey P. Shannon, Sara Assadian, Linda Lapp, Rithika Nair, Tao Huan, Nilu Partovi, Mustafa Toma, Scott J. Tebbutt

**Affiliations:** a Prevention of Organ Failure (PROOF) Centre of Excellence, Providence Research, Providence Healthcare, Vancouver, British Columbia, Canada; b Centre for Heart Lung Innovation, St. Paul's Hospital, University of British Columbia, Vancouver, British Columbia, Canada; c Department of Medicine, University of British Columbia, Vancouver, British Columbia, Canada; d Department of Chemistry, University of British Columbia, Vancouver, British Columbia, Canada; e Pharmaceutical Sciences, Vancouver General Hospital, Vancouver, British Columbia, Canada; f Faculty of Pharmaceutical Sciences, University of British Columbia, Vancouver, British Columbia, Canada; g Division of Cardiology, Providence Health Care, St. Paul's Hospital, University of British Columbia, Vancouver, British Columbia, Canada

**Keywords:** heart transplantation, acute cellular rejection, tacrolimus, endomyocardial biopsy, immunosuppression

## Abstract

**Background:**

Acute cellular rejection (ACR) is a common complication following heart transplantation (HTx). This study examined the association between tacrolimus whole-blood concentrations and endomyocardial biopsy (EMB)-proven ACR in adult HTx recipients.

**Methods:**

We conducted a retrospective analysis of 41 adult HTx recipients enrolled in the HEARTBiT study at St. Paul’s Hospital (Vancouver, Canada) between August 2018 and February 2020. A total of 315 EMB visits were analyzed and matched with tacrolimus whole-blood trough concentrations measured within ±1 day using liquid chromatography-tandem mass spectrometry. Patients were stratified into 2 post-transplant intervals: 0 to 90 days and 91 to 180 days, based on BC Clinical Guidelines for Transplant Medications for target tacrolimus levels.

**Results:**

During the first 90 days post transplant, tacrolimus concentrations were significantly lower in 2R rejection episodes compared to both 0R (*p* = 0.006) and 1R (*p* = 0.013) groups. No significant differences in tacrolimus levels were observed beyond 90 days. In a linear mixed effects model adjusting for time post transplant (days) and tacrolimus dose, 2R rejection remained independently associated with lower tacrolimus concentrations (−2.73 µg/ml; *p* = 0.021), despite slightly higher dosing at those visits (+0.10 mg/d; *p* = 0.047). Clinical review confirmed no concurrent cytomegalovirus infections or major changes in other immunosuppressive therapies.

**Conclusions:**

Lower tacrolimus concentrations during moderate ACR episodes were not attributable to underdosing or clinical confounders, suggesting the role of altered pharmacokinetics or patient-specific factors. Taken together, our results emphasize the clinical relevance of tailoring tacrolimus targets to individual pharmacokinetics, especially in early-phase post-transplant care.

## Background

Cardiac allograft rejection remains a leading cause of morbidity and mortality following heart transplantation (HTx), despite advances in immunosuppressive therapy.^1^ Among the various forms of rejection, acute cellular rejection (ACR) is the most common in the early post-transplant period and poses a significant threat to graft function. Routine surveillance for ACR typically relies on serial endomyocardial biopsies (EMBs), an invasive and resource-intensive procedure that remains the gold standard for diagnosis. Tacrolimus, a calcineurin inhibitor, is the cornerstone of maintenance immunosuppression in HTx recipients. However, its narrow therapeutic index and high interindividual pharmacokinetic variability complicate dose optimization. Subtherapeutic levels are associated with increased risk of rejection, while supratherapeutic levels can lead to nephrotoxicity and other adverse effects.^2^ Emerging evidence, particularly from kidney transplantation studies, highlights the importance of maintaining stable, therapeutic tacrolimus concentrations to reduce rejection episodes and improve long-term graft outcomes.^3^ In HTx, the relationship between tacrolimus blood levels and biopsy-proven ACR remains incompletely understood, especially within the early post-transplant period when rejection risk is highest.^1^, ^4^ Recent longitudinal data confirm that the majority of clinically relevant rejection episodes occur within the first 6 months after heart transplant, supporting the critical importance of early immunosuppressive surveillance.^5^ This study aimed to evaluate the association between tacrolimus whole-blood trough concentrations and the presence of ACR in adult HTx recipients, with a focus on the first 180 days post transplant.

## Methods

### Study design and population

This retrospective, single-center study included all adult heart transplant recipients enrolled in the HEARTBiT study (ClinicalTrials.gov Identifier: NCT03575910) at St. Paul’s Hospital (Vancouver, BC, Canada) between August 2018 and February 2020. The HEARTBiT study is a prospective cohort evaluating a novel minimally invasive transcriptomic blood biomarker for managing acute cardiac allograft rejection.

### Inclusion and exclusion criteria

Eligible participants were adults (≥18 years) who had undergone HTx and were receiving maintenance immunosuppressive therapy that included tacrolimus.^6^, ^7^ For inclusion in this analysis, whole-blood samples with tacrolimus trough concentrations measured within ±1 day of EMB collection were required. Samples were excluded if biopsy tissue was insufficient for histologic assessment, if the diagnosis was isolated antibody-mediated rejection (AMR), or if the sample was collected beyond 180 days post transplant.

### Immunosuppressive protocols

Immunosuppressive protocols included tacrolimus corticosteroids and mycophenolate mofetil, in accordance with British Columbia’s Clinical Guidelines for Transplant Medications and the International Society for Heart and Lung Transplantation (ISHLT) recommendations.8., 9 Tacrolimus was initiated orally at 0.1 mg/kg/d divided into 2 doses every 12 hours. Dose adjustments were guided by whole-blood trough concentrations per local therapeutic targets.^8^ For corticosteroids, all patients received intravenous methylprednisolone intraoperatively and postoperatively on the day of transplant. This was followed by an oral prednisone taper, starting at approximately 40 mg/d and down to 20 mg/d at the time of the first scheduled biopsy. Prednisone was weaned based on biopsy findings and clinical status, with most patients discontinuing corticosteroids by week 16 post transplant. For antimetabolite therapy, most patients received mycophenolate mofetil at a standard dose of 1,000 mg orally twice daily. Dose reductions were made in response to gastrointestinal side effects, leukopenia, or increased infection risk. Mycophenolate sodium, sirolimus, and azathioprine were not routinely used.^8^

### Therapeutic drug monitoring of tacrolimus

Tacrolimus trough concentrations were quantified using liquid chromatography-tandem mass spectrometry on EDTA-treated whole-blood samples collected on the day of routine monitoring (±1 day of biopsy). These measurements were not mandated by the HEARTBiT protocol and reflect real-world therapeutic drug monitoring practices. Target trough levels were 9 to 12 ng/ml during the first 3 months post-HTx and 8 to 9 ng/ml between 3- and 6-month post-HTx, based on British Columbia’s Clinical Guidelines for Transplant Medications.^8^ Accordingly, recipients were stratified into 2 intervals for analysis: early post transplant (0-90 days) and late post transplant (91-180 days). This time window reflects changes in immunologic risk and immunosuppressive intensity in local clinical practice and aligns with guideline-defined therapeutic targets.

### Endomyocardial biopsy protocol

Biopsies were performed at scheduled intervals or in response to clinical suspicion of rejection, following institutional protocol. According to the HEARTBiT protocol, EMBs were performed at predefined intervals at weeks 2, 3, 4, 6, 8, 10, 12, 16, 18, 22, 30, 38, and 52 post transplant, reflecting the institutional standard of care at the time of enrollment. In this cohort, all 2R rejection episodes were detected through protocol-scheduled biopsies, and all patients adhered to the scheduled biopsy timeline. Tacrolimus blood levels were measured as part of routine clinical care and were not mandated by the HEARTBiT study protocol. Over 90% of the samples included in this analysis were collected on the same day as the corresponding EMB to ensure close temporal alignment for exposure-response evaluation. Biopsies were interpreted according to the ISHLT 2005 revised grading system for ACR.^10^ In this study cohort, all 41 patients adhered to the scheduled biopsy timeline. All 2R rejection episodes were identified through protocol-scheduled EMBs rather than clinically indicated biopsies. No ISHLT grade 3R rejection episodes occurred during the 180-day follow-up period.

### Cytomegalovirus management

Patients were managed according to ISHLT Guidelines and British Columbia’s provincial protocols for transplant cytomegalovirus (CMV) infection monoprophylaxis.8., 9 CMV prophylaxis with oral valganciclovir (900 mg once daily, adjusted for renal function) was administered for 3 months in cases of donor-positive/recipient-negative (D+/R−) mismatch and following corticosteroid or antithymocyte globulin therapy for acute rejection. Outside of prophylaxis periods, routine CMV surveillance was performed using plasma CMV DNA PCR, with preemptive treatment initiated if viral load thresholds were exceeded or clinical symptoms developed. Intravenous ganciclovir was used for moderate-to-severe CMV disease or intolerance to oral therapy.^8^

### Statistical analysis

Descriptive statistics were used to summarize baseline characteristics. Continuous variables were reported as medians with interquartile ranges or means with standard deviations, as appropriate. Categorical variables were presented as frequencies and percentages. Tacrolimus concentrations across rejection grades were compared using 1-way analysis of variance followed by Tukey’s post-hoc tests for pairwise comparisons. Statistical comparisons were stratified by early (0-90 days) and late (91-180 days) post-transplant intervals. To evaluate the independent association between tacrolimus blood levels and ACR, 4 modeling approaches were used. Interval-specific linear regression models assessed the relationship between tacrolimus trough concentrations and ACR status (2R vs 0R/1R) within the early (0-90 days) and late (91-180 days) post-transplant periods. Each rejection episode was matched to a single tacrolimus measurement. Linear mixed effects models were constructed for the full cohort to evaluate the overall association between tacrolimus levels and ACR status across repeated measurements. These models included a random intercept for each patient and fixed effects for time post transplant (days), daily tacrolimus dose (mg/day), and rejection grade. As sensitivity analyses, logistic regression was used to evaluate the association between tacrolimus concentration and 2R rejection within 90 days post transplant, adjusting for dose and time post transplant. Finally, we used a Cox proportional hazards model to assess rejection-free survival within 90 days post transplant, stratified by tacrolimus concentration categories. All statistical analyses were performed using R (version 4.4.3). A 2-sided *p*-value <0.05 was considered statistically significant. This study was approved by the University of British Columbia Research Ethics Board and conducted in accordance with the Declaration of Helsinki and the Tri-Council Policy Statement (TCPS 2).

## Results

**Figure 1:**
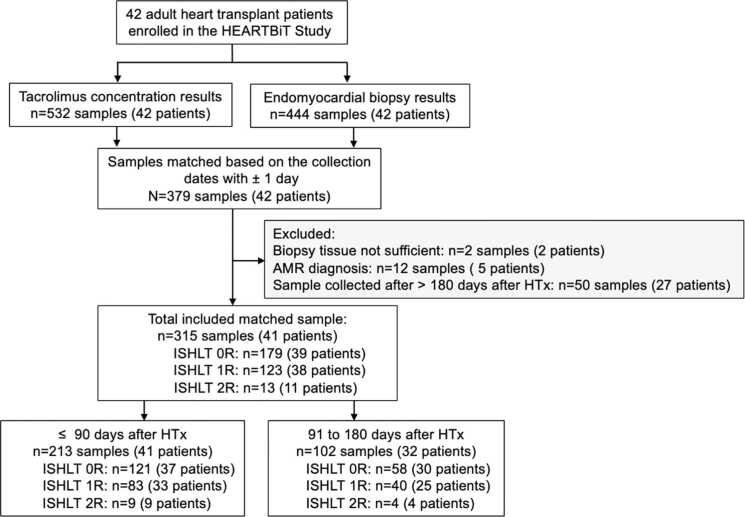
Selection process for blood samples and matched endomyocardial biopsies to confirm rejection status. The reference standard for acute cellular rejection status was determined by the histology-based rejection grade, following ISHLT 2005 guidelines (0R—no rejection, 1R—mild rejection, 2R—moderate rejection, 3R—severe rejection). Heart tissue for this assessment was obtained from an endomyocardial biopsy (EMB) performed within ±1 day of the blood collection for tacrolimus concentration testing. Histopathology grades were assessed by pathologists. Abbreviations: AMR, antibody-mediated rejection; HTx, heart transplantation; ISHLT, International Society for Heart and Lung Transplantation.

A total of 315 EMBs were analyzed from 41 adult HTx recipients (28 male, 13 female; median age: 60 years, interquartile range: 14) across thirteen scheduled follow-up visits during the first 180 days post transplantation (Figure 1). Patient demographics and baseline clinical characteristics are presented in Table 1. Thirteen cases of ACR were documented, with 9 occurring within the first 90 days and 4 between days 91 and 180 post transplant. No grade 3R ACR episodes were observed, and no 3R cases had matched tacrolimus trough levels available for analysis. All 41 patients completed the scheduled protocol biopsy visits without deviation during the 180-day follow-up. None of the 2R episodes prompted a repeat biopsy within 48 hours; patients resumed protocol EMBs after initiating antirejection therapy.

**Table 1:** Baseline Demographics Characteristics With Concurrent Tacrolimus Testing and Endomyocardial Biopsy After Heart Transplantation

Variable		Full dataset	≤90 d	91-180 d
Total patients	N	41	41	32
Age	Median (IQR)	60 (14)	60 (14)	60 (16)
Sex	Male, *n* (%)	28 (68.3)	28 (68.3)	22 (68.8)
	Female, *n* (%)	13 (31.7)	13 (31.7)	10 (31.2)
Total number of matched samples 0R 1R 2R		315 179 123 13	213 121 83 9	102 58 40 4
Tacrolimus testing the day before the biopsy				
Number of matched samples 0R 1R 2R		19 10 5 4	17 9 4 4	2 1 1 0
Same-day tacrolimus testing during the biopsy				
Number of matched samples 0R 1R 2R		284 163 113 8	184 106 74 4	100 57 39 4
Tacrolimus monitoring the day after the biopsy				
Number of matched samples 0R 1R 2R		12 6 5 1	12 6 5 1	0 0 0 0

Abbreviation: IQR, interquartile range.

### Tacrolimus trough concentration and ACR status

**Figure 2:**
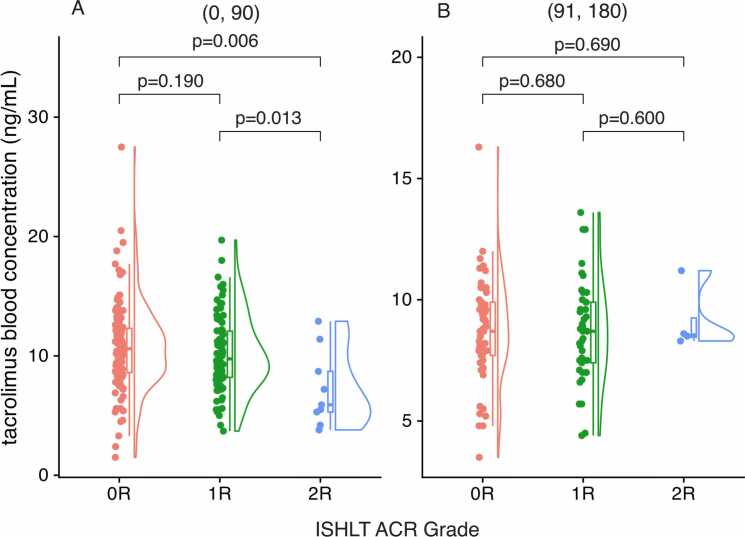
Analysis of blood tacrolimus trough concentrations across acute cellular rejection (ACR) grades. (A) Within 90 days post transplant, tacrolimus levels were significantly lower in 2R rejection compared to 0R and 1R. (B) Between 91 and 180 days, no significant differences were observed. Tacrolimus levels were measured in EDTA-treated whole blood using liquid chromatography-tandem mass spectrometry (AB Sciex API 5,000 coupled with a Shimadzu 20AC MPX system), the routine platform for therapeutic drug monitoring at St. Paul’s Hospital, Vancouver, Canada.

Tacrolimus blood levels were significantly associated with ACR grade in the early post-transplant period. Between days 0 and 90, mean tacrolimus concentrations were significantly lower in visits corresponding to 2R rejection compared with 0R (*p* = 0.006) and 1R (*p* = 0.013) groups (Figure 2A). No significant differences in tacrolimus concentrations were detected between rejection grades during the 91- to 180-day period (Figure 2B). These trends were corroborated by visual inspection (Figure S1) and supported by linear regression modeling (Table S1). In interaction models, 2R episodes within the first 90 days were associated with a 3.28 µg/ml lower tacrolimus concentration (95% CI: −5.33 to −1.23; *p* = 0.002) compared with non-2R visits. No significant difference was observed beyond 90 days (*p* = 0.765). As a sensitivity analysis, multivariable logistic regression was performed within the first 90 days post transplant to evaluate the independent association between tacrolimus concentrations and 2R rejection, adjusting for daily dose and time since transplant. Lower tacrolimus concentrations were independently associated with higher odds of 2R rejection (OR 0.74 per 1 ng/ml increase, 95% CI: 0.58-0.95; *p* = 0.018), indicating that decreased exposure was linked to increased rejection risk (Table S2).

To further assess the clinical relevance of tacrolimus concentration thresholds, levels were categorized as low, target, or high based on BC Clinical Guidelines.^8^ In the early post-transplant period (0-90 days), 77.8% (7/9) of 2R rejection episodes occurred during periods of tacrolimus levels <9 ng/ml, compared to 31.4% (38/121) of 0R and 37.3% (31/83) of 1R episodes (Tables S3 and S4). The association between categorical tacrolimus levels and rejection grade approached statistical significance (*p* = 0.066). In contrast, no significant differences were observed during the 91- to 180-day interval (*p* = 0.196).

### Tacrolimus dose and ACR

**Figure 3:**
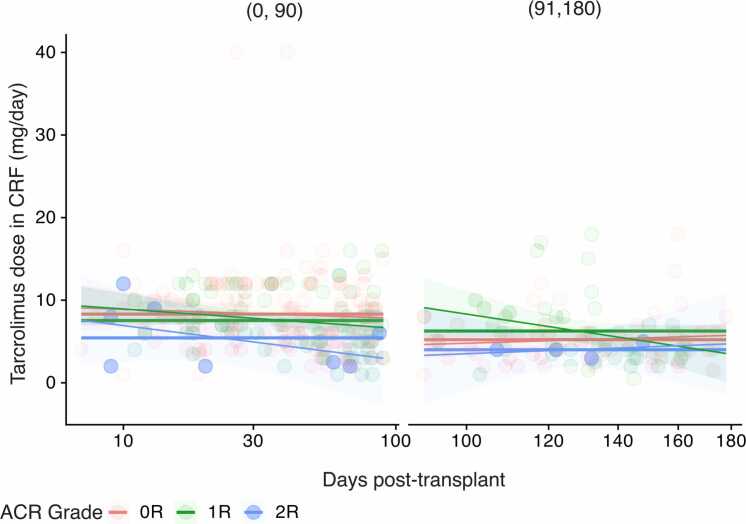
Tacrolimus daily doses (mg/day) by ACR grade over time in 2 post-transplant intervals (0-90 and 91-180 days). Each point represents an individual EMB visit, color-coded by ACR grade (0R = red, 1R = green, 2R = blue). Solid lines indicate local regression trends with 95% confidence intervals. Notably, 2R rejection episodes, particularly in the early period, occurred despite comparable or higher tacrolimus doses, suggesting that lower drug concentrations were not due to intentional underdosing. Note: Tacrolimus dosing data were unavailable for one of the 13 2R episodes within 90 days post transplant. ACR, acute cellular rejection; CRF, case report form.

Tacrolimus dosing was evaluated to determine whether reduced levels in 2R rejection reflected dose adjustments. Linear mixed effects modeling incorporating a random intercept for participants and fixed effects for time, dose, and rejection grade showed that 2R visits remained significantly associated with lower tacrolimus concentrations (−2.73 µg/ml; 95% CI: −5.07 to −0.38; *p* = 0.021) even after adjusting for dosing and time since transplant. Interestingly, tacrolimus dosing was modestly but significantly higher during 2R visits (+0.10 mg/d; 95% CI: 0.00-0.21; *p* = 0.047), suggesting that dose differences do not account for the observed trough level reductions (Table S5). This finding is illustrated in Figure 3, which shows that tacrolimus dosing did not decrease over time in 2R visits and was modestly higher compared to 0R and 1R episodes, particularly in the early post-transplant period. A review of clinical records confirmed that no cases of CMV infections were present at the time of any biopsy-proven 2R rejection episodes. No major changes to maintenance immunosuppressive therapy were noted during these events. These findings support the conclusion that decreased tacrolimus exposure during moderate rejection was not attributable to intentional immunosuppression reduction or infectious complications.

### Rejection-free survival analysis

To further evaluate the association between early tacrolimus exposure and moderate rejection, we performed an exploratory Kaplan-Meier analysis stratified by trough concentrations during the first 90 days post transplant. When using the 9 ng/ml threshold recommended by British Columbia and institutional guidelines, rejection-free survival did not differ significantly between groups (log-rank *p* = 0.095; Figure S2A). In contrast, applying a lower threshold of 8 ng/ml revealed a statistically significant difference (log-rank *p* = 0.021; Figure S2B).

### Hematological and biochemical correlates

Comparative laboratory analyses across rejection grades (Tables S6 and S7) revealed nonsignificant trends toward lower hemoglobin and red blood cell counts in 2R episodes. These findings, while not statistically significant, are consistent with hypotheses suggesting hematocrit and erythrocyte distribution may influence whole-blood tacrolimus concentrations.

## Discussion

In this retrospective cohort of adult heart transplant recipients, we investigated the association between tacrolimus blood concentrations and biopsy-confirmed ACR during the first 180 days post transplant. By analyzing 315 matched tacrolimus blood and EMB pairs, we found that lower tacrolimus blood levels were significantly associated with higher rejection grades, particularly within the first 90 days post transplant. The concentration of acute rejection events within the early post-transplant period, as demonstrated in a recent 36-year retrospective EMB study,^5^ highlights the clinical value of early immunosuppressive monitoring strategies and validates the time frame selected in our analysis.

Our study highlights the clinical variability in tacrolimus exposure at the time of rejection and underscores the importance of individualized monitoring. First, we ensured tight temporal alignment between tacrolimus level measurements and EMB pathology grading, with over 90% of samples collected on the same day. Second, we explored whether the lower tacrolimus concentrations observed in moderate rejection (2R) were due to differences in dosing or other confounders. Using a linear mixed effects regression model adjusting for time post transplant and daily dose, we found that 2R rejection episodes were still associated with significantly lower tacrolimus levels (−2.73 µg/ml; *p* = 0.021), despite slightly higher prescribed doses at those visits (+0.10 mg/d; *p* = 0.047). Clinical chart review further confirmed the absence of CMV infections or changes in other immunosuppressive agents, excluding these as plausible confounders. As illustrated in Figure S1, tacrolimus dosing did not decrease over time in 2R visits; in fact, early 2R episodes often occurred at similar or higher doses compared to 0R and 1R visits. This pattern suggests that biological variability, such as differences in drug absorption, metabolism, or distribution, rather than underdosing, may contribute to underexposure in certain individuals. Prior studies have shown that predose tacrolimus levels do not reliably correlate with administered dose or predict rejection risk, underscoring the limitations of trough-based monitoring.^11^ Even under standardized dosing protocols, individual variability in differences in absorption, metabolism, and distribution may lead to underexposure and insufficient immunosuppression.^12^ One possible explanation is genetic polymorphisms. For example, pediatric heart transplant recipients with the CYP3A5 *1/*3 expressor genotype require significantly higher doses to achieve therapeutic levels.^13^ Similar associations have been reported in adults, where CYP3A5 and CYP3A4 expression status influences dose-adjusted tacrolimus exposure.^14^ Although our study lacked pharmacogenetic data, these findings support the future integration of genotype-guided dosing to improve therapeutic precision.

To complement continuous-level analysis, we also examined tacrolimus concentrations by categorical ranges. In the early period, 78% of 2R episodes occurred during levels <9 ng/ml, compared to 31% in 0R and 37% in 1R episodes. While the association did not meet conventional thresholds for statistical significance, the consistent trend across both chi-square and Fisher’s exact tests suggests a hypothesis-generating relationship that warrants validation in larger studies. This trend was further supported by exploratory Kaplan-Meier analyses, where trough levels <8 ng/ml were significantly associated with shorter rejection-free survival. These findings reinforce the hypothesis that subtherapeutic exposure may increase early rejection risk. These exploratory findings suggest that tacrolimus levels below 8 ng/ml may be associated with an increased risk of moderate (2R) rejection in the early post-transplant period. Although institutional and provincial guidelines recommend a threshold of 9 ng/ml, the statistically significant survival difference observed with an 8 ng/ml cutoff may indicate potential value in tighter exposure control. However, given the post hoc nature of this analysis, these results should be interpreted with caution and require prospective validation in larger, multicenter cohorts. These findings are consistent with evidence from renal transplantation, where trough tacrolimus levels have been shown to correlate with systemic exposure and acute rejection risk. For instance, a pooled analysis of randomized controlled trials demonstrated that tacrolimus levels <4 ng/ml were associated with a 6.33-fold increased risk of biopsy-proven rejection in kidney transplant recipients during the first-year post transplant.^15^ Such data support the notion that maintaining adequate trough levels is critical, although the optimal threshold may vary across populations and organ types. Notably, approximately 33% of biopsy-matched samples with tacrolimus levels <9 ng/ml did not exhibit 2R rejection. This variability underscores interindividual differences in rejection susceptibility, reinforcing the rationale for personalized therapeutic targets rather than fixed thresholds. Even with dose adjustment and tight sampling alignment, variability may stem from unmeasured factors such as adherence, metabolism, or hematocrit-related distribution. These findings support the need for individualized immunosuppressive strategies, especially during early high-risk periods. Interestingly, 63% of 1R episodes occurred at tacrolimus levels ≥9 ng/ml, suggesting a weaker and less consistent association between 1R and drug exposure compared to 2R. This likely reflects the biological heterogeneity of 1R, which has been shown to encompass a spectrum of immune activity and molecular phenotypes not necessarily captured by histologic grading. Previous studies have demonstrated that 1R biopsies may include both quiescent and active forms of rejection with distinct gene expression profiles and prognostic implications.^16^ This observation aligns with current clinical practice, where isolated 1R is often subclinical and managed conservatively unless persistent or accompanied by graft dysfunction.^17^ In contrast, the more consistent relationship between lower tacrolimus levels and 2R rejection highlights the importance of maintaining therapeutic drug exposure in the early post-transplant period. Our findings underscore the need for individualized monitoring strategies and support future studies investigating immunologic or molecular subtypes of 1R with differing clinical relevance. This may partly explain the weaker and inconsistent association between 1R and tacrolimus levels observed in our cohort.

To our knowledge, this is among the first studies in adult heart transplant recipients to demonstrate a statistically significant association between moderate ACR (2R) and lower tacrolimus concentrations, using tightly time-matched blood and biopsy samples. Unlike previous studies that examined long-term trough variability or relied on broader post-transplant intervals, our design allowed for real-time assessment of tacrolimus exposure at the exact time of histologically confirmed rejection. This approach highlights limitations of standard dosing protocols without individualized monitoring and illustrates the importance of contemporaneous exposure-response characterization.

Our observations are consistent with previous findings in solid organ transplantation. For example, Gueta et al^18^ reported that higher variability in tacrolimus trough levels, even with similar mean levels, was associated with increased risk of rejection beyond the first-year post-heart transplant. In our recent metabolomics biomarker study using plasma samples from the HEARTBiT cohort, we observed no significant differences in plasma tacrolimus concentrations among 0R, 1R, and 2R rejection grades (*n* = 16, each group).^19^ In contrast, the present analysis of whole-blood tacrolimus revealed a significant association between lower concentrations and 2R rejection. Our results are consistent with previous studies showing that tacrolimus is predominantly distributed within erythrocytes (up to 85%-95%) and that whole-blood levels more accurately represent systemic exposure relevant to immunosuppressive efficacy.^2^ This is relevant because whole-blood levels incorporate both plasma and intracellular erythrocyte-bound fractions, which may better reflect the pharmacologically active pool. Plasma concentrations reflect only the unbound or minimally bound fraction and may not reliably indicate intracellular activity at the site of T-cell suppression. These findings emphasize the choice of biological matrix (plasma, whole blood, or intracellular) can significantly influence the interpretation of tacrolimus exposure and its association with immunologic responses during acute rejection episodes. Differences in sample matrices, handling protocols, or timing may also contribute to discordant findings and warrant further methodological standardization.^20^

We also explored hematologic factors that may influence tacrolimus distribution. Although hemoglobin and red blood cell counts were not statistically different between rejection groups, they trended lower in 2R episodes. This raises the possibility that erythrocyte binding variability may contribute to lower whole-blood concentrations despite adequate dosing. Prior work has shown that hematocrit, inflammation, and intracellular distribution can impact tacrolimus exposure.^21^, ^22^, ^23^ Even with protocol-based dosing, interindividual differences in absorption, metabolism, and distribution may lead to underexposure in vulnerable patients. Our data support emerging strategies for individualized tacrolimus monitoring, particularly in the early post-transplant period when patients often experience hemodynamic instability, bleeding, transfusions, or systemic inflammation.^2^ Future protocols might incorporate hematocrit-adjusted or intracellular tacrolimus monitoring, or genotype-guided dosing, for example, accounting for CYP3A5 polymorphisms, which have been shown to affect tacrolimus metabolism.^24^ Despite adequate dosing, patients with faster metabolism or altered distribution may remain underexposed during periods of heightened immunologic risk. These insights support the use of individualized, exposure-guided immunosuppressive strategies in HTx.

While our primary analysis focused on tacrolimus concentrations within ±1 day of EMB to ensure close alignment with rejection grading, we recognize that trends in drug exposure over preceding days or weeks may offer additional insight. However, due to the retrospective nature of the study and inconsistent availability of pre-EMB levels, particularly in early post-transplant episodes, we were unable to evaluate longitudinal patterns. Our aim was to characterize the pharmacologic milieu at the time of histologically confirmed rejection, though we acknowledge that earlier tacrolimus exposure, particularly in the preceding week, may better reflect causative underexposure. Same-day levels do not inform immediate clinical decision-making due to biopsy result delays but offer mechanistic insight into the exposure-response relationship. This underscores the need for future studies with standardized prebiopsy sampling intervals, more frequent or point-of-care tacrolimus monitoring, and genotype-informed protocols to optimize exposure and reduce rejection risk.

Our study has several limitations. It is a retrospective, single-center study with a relatively small number of moderate rejection (2R) episodes, which may limit generalizability. Selection bias may be present due to the inclusion of patients with complete matched data. Selection bias and timing variability in sample collection may contribute to exposure misclassification, although over 90% of blood-biopsy pairs were collected on the same day. Unmeasured confounders such as genetic polymorphisms, drug absorption, or adherence may also affect tacrolimus levels. We did not measure unbound or intracellular tacrolimus concentrations, nor did we assess pharmacogenetic variability. Despite these limitations, the use of tightly matched blood-biopsy pairs and multivariable adjustment strengthen the reliability of our findings.

## Conclusion

Our findings highlight the critical importance of maintaining adequate tacrolimus exposure in the early post-transplant period and support individualized immunosuppressive strategies. The observed association between subtherapeutic trough levels and moderate rejection underscores the potential value of tighter exposure control, particularly during periods of heightened immunologic risk. Future prospective studies incorporating genotype-guided dosing, hematocrit-adjusted monitoring, and real-time pharmacokinetic assessment are warranted to refine tacrolimus management and improve long-term transplant outcomes.

## Disclosure Statement

The authors thank the patients who generously donated their samples and data for this study, as well as, Ms Rong Yi, Ms Laura Burns, and their colleagues in the Clinical Chemistry Laboratory at St. Paul’s Hospital, Vancouver, for their support with clinical testing. This study was funded by the Canadian Institutes for Health Research, Genome British Columbia, Michael Smith Health Research BC, Providence Research, and the Health Innovation Funding Investment Awards from the University of British Columbia.

None of the authors have any financial relationships with commercial entities that have an interest in the subject of the presented manuscript, nor do they have any conflicts of interest to disclose.

## Declaration of Competing Interest

The authors declare the following financial interests/personal relationships, which may be considered as potential competing interests: Chengliang Yang and Scott J. Tebbutt report 2 grants from the Canadian Institutes of Health Research (grant numbers 177747 and 202409PJT), awarded to their institution to support research conducted in Canada.

## Footnotes

Appendix A Supplementary data associated with this article can be found in the online version at doi:10.1016/j.jhlto.2025.100373.
