# Supplementary material for: Association between tacrolimus blood levels and biopsy-proven acute cellular rejection in adult heart transplant recipients
Source: JHLT Open. 2025 Aug 19;10:100373. doi: 10.1016/j.jhlto.2025.100373 (PMC12444152; doi:10.1016/j.jhlto.2025.100373)
Supplement: Supplementary file 2 — Supplementary material [file mmc2.docx]

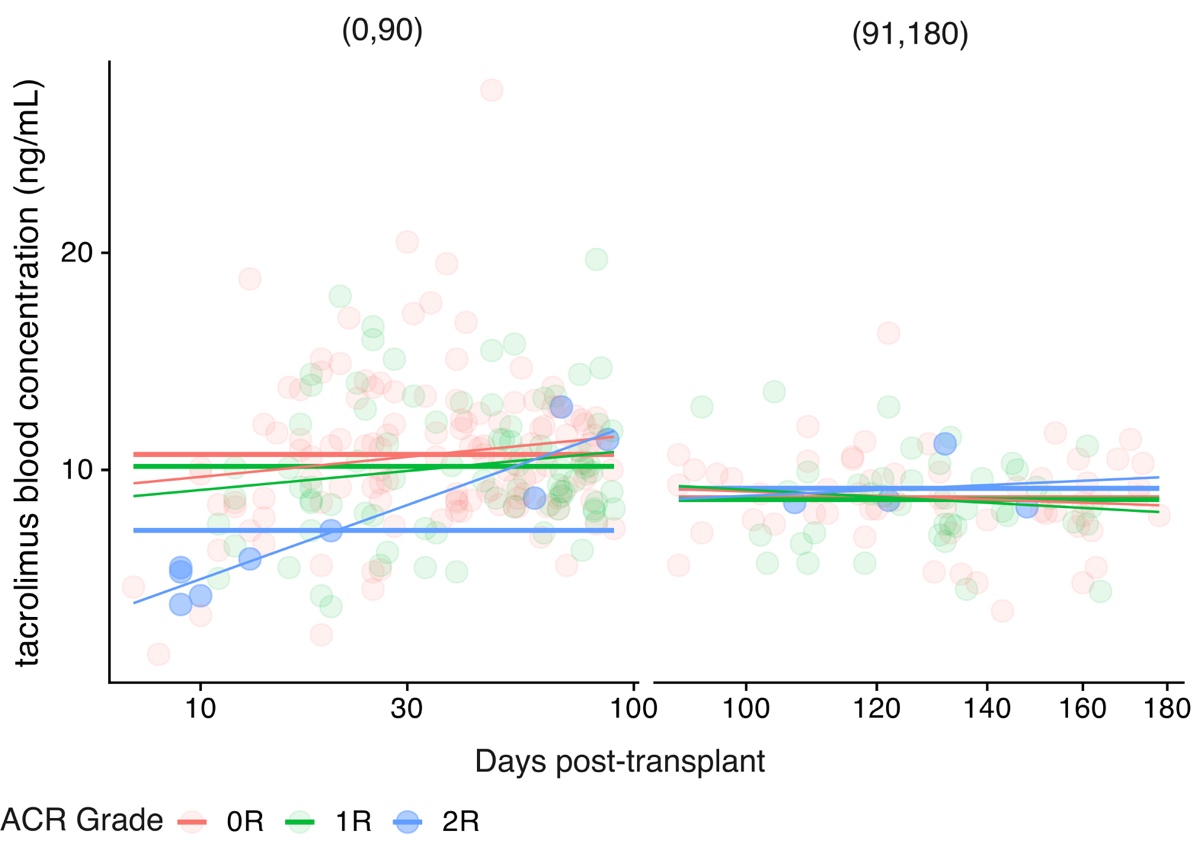


**Supplementary figure 1.** Tacrolimus trough concentrations (ng/mL) over time by ACR grade in two post-transplant intervals (0–90 and 91–180 days). Each point represents a biopsy visit with a corresponding tacrolimus measurement. Solid lines show local regression trends with 95% confidence intervals. Notably, 2R rejection episodes during the early post-transplant period occurred at lower tacrolimus concentrations than 0R or 1R visits, consistent with our regression analyses. This difference was not evident in the later period. ACR, acute cellular rejection.


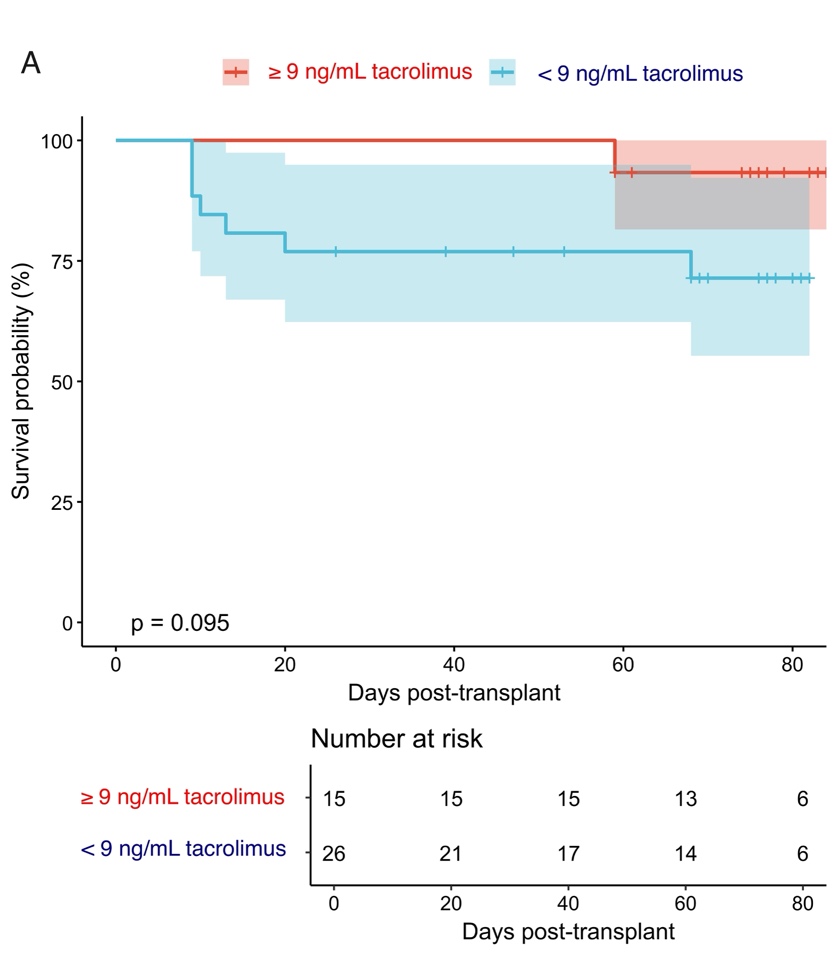

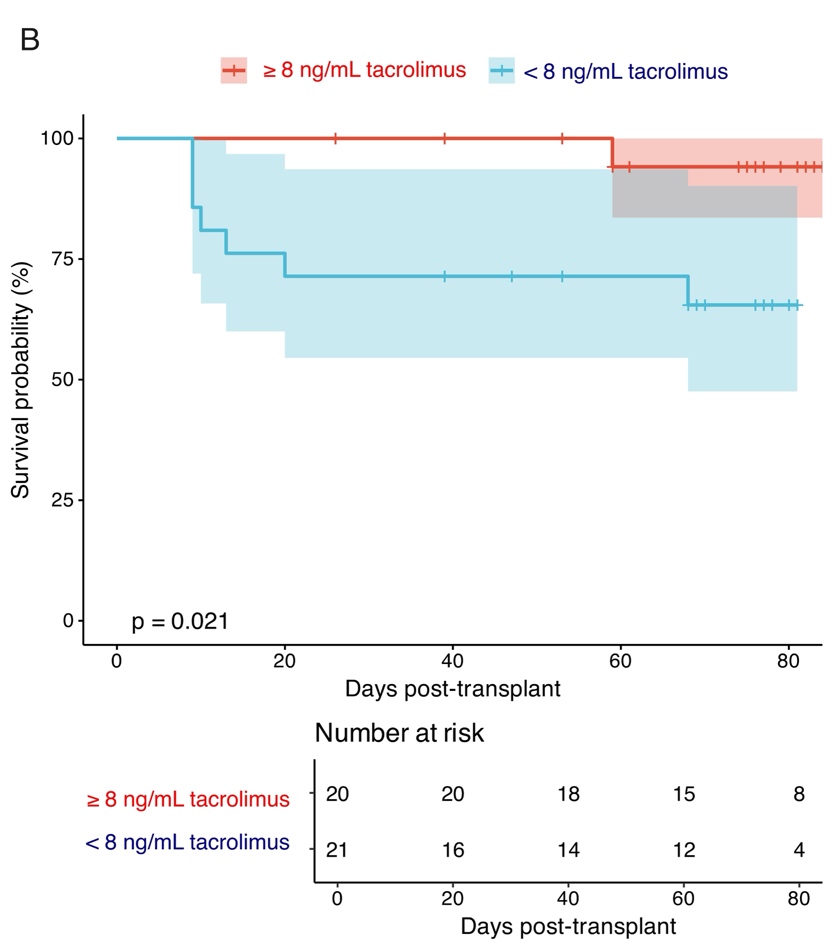


**Supplementary Figure 2. Rejection-free survival by early post-transplant tacrolimus trough concentration.** A. Kaplan-Meier analysis of rejection-free survival within 90 days post-transplant, stratified by tacrolimus concentration using a 9 ng/mL threshold. Patients with trough concentration in the <9 ng/mL group exhibited a trend toward shorter time to moderate rejection (2R), although this difference did not reach statistical significance (log-rank *p* = 0.095). B. Kaplan–Meier analysis using an 8 ng/mL threshold. Patients with trough concentrations <8 ng/mL experienced significantly shorter time to moderate rejection (2R) compared to those with levels ≥8 ng/mL (log-rank *p* = 0.021). Shaded areas represent 95% confidence intervals. Thresholds were selected for exploratory analysis based on observed distribution and institutional tacrolimus targets during the study period (9-12 ng/mL). These exploratory findings should not be interpreted as evidence to revise current therapeutic targets but rather as preliminary signals warranting further validation.
